# Robust Cylindrical Panorama Stitching for Low-Texture Scenes Based on Image Alignment Using Deep Learning and Iterative Optimization

**DOI:** 10.3390/s19235310

**Published:** 2019-12-02

**Authors:** Lai Kang, Yingmei Wei, Jie Jiang, Yuxiang Xie

**Affiliations:** College of Systems Engineering, National University of Defense Technology, Changsha 410073, China; lkang.vr@gmail.com (L.K.); yxxie@nudt.edu.cn (Y.X.)

**Keywords:** cylindrical panorama, low-texture environments, convolutional neural network (CNN), robust image alignment, sub-pixel optimization

## Abstract

Cylindrical panorama stitching is able to generate high resolution images of a scene with a wide field-of-view (FOV), making it a useful scene representation for applications like environmental sensing and robot localization. Traditional image stitching methods based on hand-crafted features are effective for constructing a cylindrical panorama from a sequence of images in the case when there are sufficient reliable features in the scene. However, these methods are unable to handle low-texture environments where no reliable feature correspondence can be established. This paper proposes a novel two-step image alignment method based on deep learning and iterative optimization to address the above issue. In particular, a light-weight end-to-end trainable convolutional neural network (CNN) architecture called ShiftNet is proposed to estimate the initial shifts between images, which is further optimized in a sub-pixel refinement procedure based on a specified camera motion model. Extensive experiments on a synthetic dataset, rendered photo-realistic images, and real images were carried out to evaluate the performance of our proposed method. Both qualitative and quantitative experimental results demonstrate that cylindrical panorama stitching based on our proposed image alignment method leads to significant improvements over traditional feature based methods and recent deep learning based methods for challenging low-texture environments.

## 1. Introduction

Panoramic images are able to provide a wide field-of-view (FOV) of a scene, which is demanded in a number of applications, such as remote sensing [1,2], environment monitoring [3,4], robot localization [5,6], autonomous transportation [7,8], etc. The ways in which panoramas are created can be divided into three categories [9]. The first category uses a special optical device (e.g., an omnidirectional sensor) to capture a single image onto which the surrounding scene is projected. Although this kind of devices are easy to use in practice, the resolution of the captured image is usually low and commonly suffers from severe distortions. The second category uses panoramic acquisition devices consisting of multiple synchronized cameras. This way is able to provide high quality panoramas but the hardware system can be more expensive than a conventional camera. The third method of panorama acquisition is using a single camera to capture a sequence of overlapping images with rotating shoot and then construct a panorama by stitching the images together, which provides a low-cost and flexible solution to generate a panoramic image with high resolution and large viewing angle.

This work follows the third way and focuses on the problem of cylindrical panorama generation from a set of images, in which an essential step is to perform accurate image alignment. In an ideal scenario where the camera undergoes pure rotation around a fixed vertical axis passing through its perspective projection center and the amount of details in the target scene is sufficiently high, the alignment of two consecutive images can be simplified to a horizontal image displacement estimation problem, which can be readily fulfilled by classic image matching algorithms based on sparse local image features [10]. However, the assumption that the rotation axis passes through the optical center is very unrealistic, thus image alignment based on horizontal tilt motion model commonly results in artifacts. In addition, the above feature based method cannot handle sparsely structured scenes lacking texture. To address the above issues, this paper proposes a robust solution to cylindrical panorama generation for low-texture environments, with explicit modeling of global illumination change and camera motion. In particular, this paper makes the following contributions:

- A novel light-weight regression convolutional neural network (CNN) architecture called ShiftNet, able to handle low-texture environments properly, is proposed to predict robust shift estimates. ShiftNet can be trained in an end-to-end fashion using a large synthetic dataset generated from a publicly available image dataset. The performance of ShiftNet is superior to recent CNN models in terms of both model size and inference accuracy.
- A global illumination invariant sub-pixel refinement algorithm is proposed to improve the accuracy of the initial estimate obtained by ShiftNet under either a strict horizontal translation motion or a translate and scale motion assumption. The refinement procedure is essential for cylindrical panorama stitching because the outputs of a CNN model are not accurate enough to produce visually pleasing results.
- Extensive experiments on a synthetic dataset rendered photo-realistic images, and real images were then tested to throughly evaluate the performance of the proposed method and comparative methods. Both qualitative and quantitative experimental results for challenging low-texture environments demonstrated significant improvements over traditional feature based methods and recent deep learning based methods.

This remainder of this paper is structured as follows. Section 2 provides a brief history of the literature on related topics regarding panorama generation. Section 3 presents details on key components of the cylindrical panorama stitching framework, including an analysis of the geometry of cylindrical projection, a new CNN architecture for initial image alignment, a sub-pixel refinement procedure to improve alignment accuracy, and also information regarding the synthetic training and test image datasets. In Section 4, we present qualitative and quantitative experimental results along with a comprehensive analysis. Finally, concluding remarks and the future work are presented in Section 5.

## 2. Related Works

By combining multiple images with overlapping FOVs, cylindrical panorama stitching provides an easy way to produce a high-resolution global image with large viewing angle [9]. The most important step in this process is to accurately align two images, which corresponds to finding a mapping between the coordinate systems of two images. In the case of cylindrical panorama construction, image alignment can be simplified as a problem of estimating one or more motion parameters under a specific geometric transformation model.

The most widely used image alignment methods are methods based on hand-crafted features. These methods first extract a set of image keypoints and compute their descriptors, and then find a set of putative feature correspondences by matching the descriptors, after which outliers in the initial correspondence set are eliminated and the geometric transformation between the two images is calculated within a RANdom Sample Consensus (RANSAC) scheme [11]. In the literature, many image feature detection and description algorithms have been proposed, among which scale-invariant feature transform (SIFT) [12], speeded up robust features (SURF) [13], oriented features from accelerated segment test (FAST) and rotated binary robust independent elementary features (BRIEF) (which is called ORB) [14], accelerated-KAZE (AKAZE) [15], and binary robust invariant scalable keypoints (BRISK) [16] are some popular ones. The most well-known feature detection and description algorithm, SIFT, is based on the difference-of-Gaussian (DoG) operator which is an approximation of Laplacian-of-Gaussian (LoG) [17]. The SURF detector is based on the determinant of Hessian Matrix and it exploits integral images to improve the computational efficiency of feature detection. The ORB algorithm is a blend of the modified FAST (features from accelerated segment test) [18] detection and direction-normalized BRIEF (binary robust independent elementary features) [19] methods. AKAZE is based on non-linear diffusion filtering like KAZE [20], however its non-linear scale spaces are constructed using a computationally efficient framework called fast explicit diffusion (FED). BRISK detects corners using the AGAST algorithm [21] and filters them with the FAST [18] corner score while searching for maxima in the scale space pyramid. The BRISK description is based on identifying the characteristic direction of each feature for achieving rotation invariance. A detailed analysis and comparison of these feature detection and description algorithms can be found in [22,23]. In addition to image alignment, there are also some other less relevant works focusing on improving the global quality of panorama images, e.g., a method (based on SIFT features) to mitigate the projective distortion produced by homography was proposed in [24], and a super-resolution algorithm for equirectangular panorama images was proposed in [25].

During the last decade, deep learning has made remarkable advances in various applications in computer vision [26,27,28,29,30], such as image categorization, image segmentation, object detection, etc. More recently, the use of CNNs to solve geometric problems has also been investigated and promising results have been reported. For example, different CNN models have been proposed to estimate a homography matrix [31,32] and fundamental matrix [33,34]. The first attempt to apply CNN to solve a two-view geometric problem was made by DeTone et al. [31], who presented an end-to-end trainable VGG-style [35] CNN architecture for estimating the relative homography between two images. The above method uses a four point homography parameterization which maps the four corners from one image into the second one. The robustness and accuracy of homography estimation can be further improved by combining CNN and photometric refinement [32].

Ranftl et al. [33] proposed an approach for robust fundamental matrix estimation from noisy data contaminated by outliers. The problem was cast as a series of weighted homogeneous least-squares problems, where robust weights are estimated using deep networks. Another work, also focused on estimating the fundamental matrix, introduces a CNN model which explicitly takes into account the mathematical properties of the fundamental matrix as a homogeneous rank-2 matrix with seven degrees of freedom [34]. Agrawal et al. [36] estimated ego-motion using a neural network as a pre-training step for high-level tasks. PoseNet [37,38] employed a convolutional network to estimate the pose of a given image for camera relocalization. The DeMoN architecture [39] provides, given two consecutive frames from a monocular camera, both an estimate of the depth of each pixel and an estimate of the motion between frames. The deep learning based methods for solving geometric problems achieve comparable or in some cases better performance compared with traditional methods based on hand-crafted images features.

Although feature based methods often provide excellent performance in cases when there are sufficient reliable features in the images, these methods often fail in low-texture environments (e.g., uniform walls, floors, and ceilings in indoor scenarios, or outdoor scenes dominated by sky and sea), where not enough distinct features can be detected and matched. The greatest advantage of deep learning based methods over traditional hand-crafted feature based methods for solving geometric problem is its robustness to sparse scene structure, illumination change, and image noise. While it is straightforward to adopt existing homography estimation CNN models for robust image alignment by adjusting the output layer, such modifications have been shown not to be able to obtain sufficiently accurate estimates for cylindrical panorama stitching. To this end, this paper proposes a novel end-to-end trainable light-weight CNN architecture called ShiftNet to estimate the initial shifts between images, which is further optimized in a sub-pixel refinement procedure based on a specified camera motion model. Details on our proposed method and experiments with different types of datasets are presented in the following.

## 3. Proposed Method

This paper proposes a robust solution to cylindrical panorama generation for low-texture environments, with explicit modeling of global illumination change and camera motion. In this section, we first present an overview of the proposed pipeline and then present details on key components in the pipeline, including the geometry of cylindrical projection, the CNN architecture for initial image alignment, the sub-pixel refinement procedure, as well as the method for generating synthetic dataset for training and testing the proposed CNN architecture.

### 3.1. Overview

**Figure 1:**
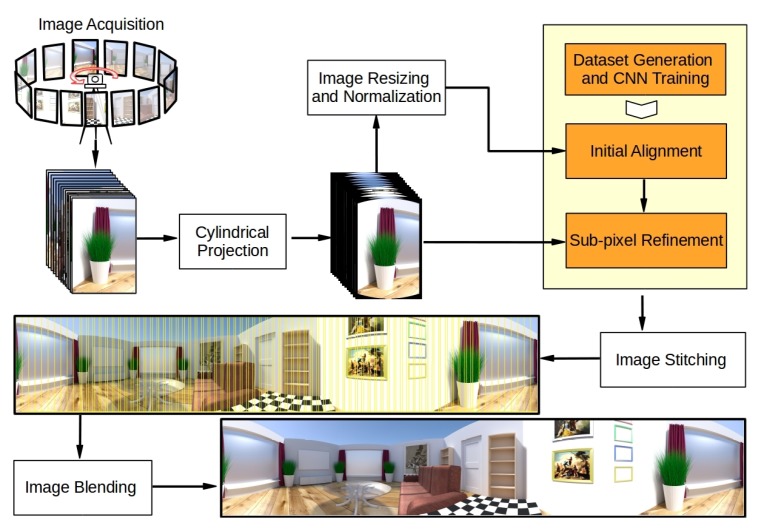
The cylindrical panorama stitching pipeline in which the main contributions of this work are highlighted (rendered photo-realistic images are taken from the publicly available dataset described in [40]). See text for details. Convolutional neural network (CNN).

As shown in Figure 1, a complete pipeline of cylindrical panorama construction for low-texture environments can be roughly divided into several steps, including image acquisition, cylindrical projection, initial alignment, sub-pixel refinement, image stitching, and image blending. The first step is to capture a sequence of overlapping images by rotating a camera around a fixed axis passing through its optical center. In cylindrical projection step, each image is projected onto the surface of a cylinder, then the corresponding cylinder surface is unrolled to make a flat plane. In the third step, an initial alignment between all pairs of consecutive images is performed using a CNN model, followed by a sub-pixel refinement procedure which improves the accuracy of alignment further in the fourth step. Finally, all images are stitched and blended together based on the refined alignment results. The following subsections focus on cylindrical projection, deep learning based initial alignment, and iterative sub-pixel refinement, which are the key components of cylindrical panorama generation. In particular, aiming at providing a reliable cylindrical panorama solution working for low-texture environments, a customized light-weight CNN model and a refinement strategy are proposed to improve the robustness and the accuracy of image alignment.

### 3.2. Cylindrical Projection

In order to construct a cylindrical panorama, all the images captured with the camera need to be projected to cylindrical coordinates, and then stitched together according to displacement between each pair of consecutive images. In the following, we presents theoretical background on cylindric projection.

**Figure 2:**
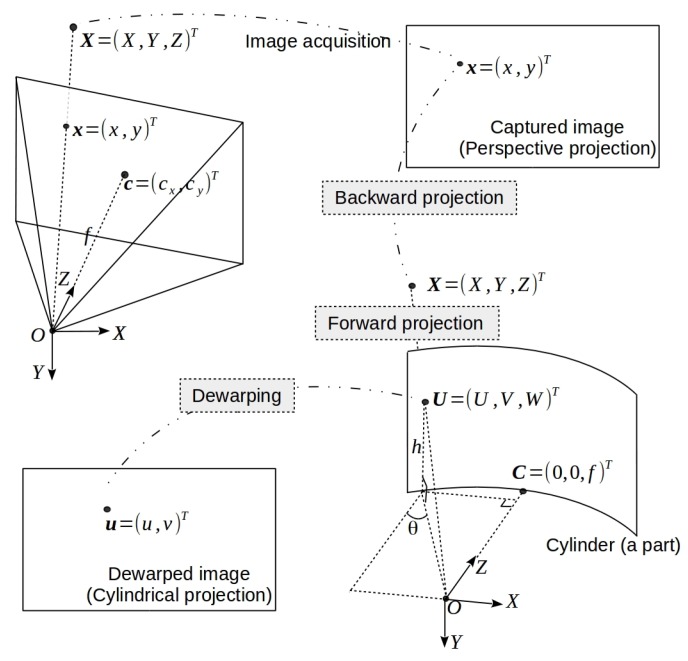
Image formation under the pinhole camera model and the workflow of cylindrical projection involving backward projection, forward projection, and image dewarping. See text for details.

In this paper, the pinhole camera model is used and the intrinsic parameters of the lens which can be obtained from calibration is assumed to be known a priori, based on which, an image can be projected to a cylindrical surface via cylindrical projection. An illustration of image formation is shown in the image at the upper-left corner of Figure 2. Let $x={\left(x,y,1\right)}^{⊤}$ be the projection of a 3D scene point $X={\left(X,Y,Z\right)}^{⊤}$ onto one image, where both x and X are represented by homogeneous coordinates. Without loss of generality, the camera is aligned to the world coordinate system, i.e., the rotation matrix and the translation vector of the camera becomes a 3×3 identity matrix I and a 3×1 zero column vector 0, respectively. Thus the camera projection process under pinhole camera model can be written as x≃K[I|0]X, where

(1) $$K=\left(\begin{array}{ccc} f & 0 & c_{x}\\ 0 & f & c_{y}\\ 0 & 0 & 1 \end{array}\right)$$

is a matrix composed by a set of intrinsic camera parameters $\left\{f,c_{x},c_{y}\right\}$. Here, *f* is the focal length of camera lens, $c={\left(c_{x},c_{y},1\right)}^{⊤}$ is the principal image point on the image plane, and the notation ≃ represents equality up to scale [41].

As illustrated in Figure 2, the cylindrical projection consists of the following three steps: backward projection to the 3D space, forward projection onto the cylindrical surface, and image dewarping to make a flat plane. Specifically, given an image point ${\left(x,y,1\right)}^{⊤}$, in the first step, the corresponding 3D scene point X can be obtained by the backward projection $X≃K^{-1}x≃{\left(\frac{x-c_{x}}{f},\frac{y-c_{y}}{f},1\right)}^{⊤}$. In the second step, the above 3D point is projected onto a cylinder surface whose center is identical to the camera projection center O (see Figure 2). The projected 3D point $U={\left(U,V,W\right)}^{⊤}$ lying on the cylinder surface can be parameterized by $\left\{\theta =\arctan\frac{x-c_{x}}{f},h=\frac{f(y-c_{y})}{\sqrt{f^{2}+{\left(x-c_{x}\right)}^{2}}}\right\}$. In the third step, the 3D point U parameterized by $\left\{\theta ,h\right\}$ is warped onto a target image plane to get the image point $u={\left(u,v,1\right)}^{⊤}$ by

(2) $$\left\{\begin{array}{l} u=c_{x}+f\frac{\theta }{2\pi }=c_{x}+\frac{f}{2\pi }\arctan\frac{x-c_{x}}{f}\\ v=c_{y}+h\;\;\;=c_{y}+\frac{f(y-c_{y})}{\sqrt{f^{2}+{\left(x-c_{x}\right)}^{2}}} \end{array}\right..$$

In this way, an image point u under cylindrical projection can be related by the corresponding image point x in the original image.

Let $u_{1}={\left(u_{1},v_{1},1\right)}^{⊤}$ and $u_{2}={\left(u_{2},v_{2},1\right)}^{⊤}$ be two final cylindric projected image points corresponding to two 3D points $U_{1}$ (parameterized by $\left\{\theta_{1},h_{1}\right\}$) and $U_{2}$ (parameterized by $\left\{\theta_{2},h_{2}\right\}$) lying on the cylinder. According to Equation (2), $u_{2}-u_{1}=\frac{f}{2\pi }\left(\theta_{2}-\theta_{1}\right)$ and $v_{2}-v_{1}=\frac{f}{2\pi }\left(h_{2}-h_{1}\right)$. In an ideal case, the camera is assumed to rotate around its *Y*-axis only, thus both $u_{1}-u_{2}$ and $v_{1}-v_{2}$ remains the same in two cylindrical projection images. In other words, the image motion corresponds to a global shift in the horizontal direction and stitching images requires estimating such a shift only. In practice, the above shift-only image alignment introduces errors due to a few factors, e.g., non-perfect camera model, rotation center not aligned to the camera projection center perfectly, low-precision of shift estimation, etc. In this case, a refinement based on a more general motion model is required.

### 3.3. Initial Alignment Using ShiftNet

As explained in the previous subsection, horizontal shift estimation is crucial for cylindrical panorama stitching. While traditional methods based on sparse features are not reliable for low-texture environments, this paper proposes a customized light-weight regression CNN architecture called ShiftNet to robustly predict shift estimates in an end-to-end fashion.

**Figure 3:**
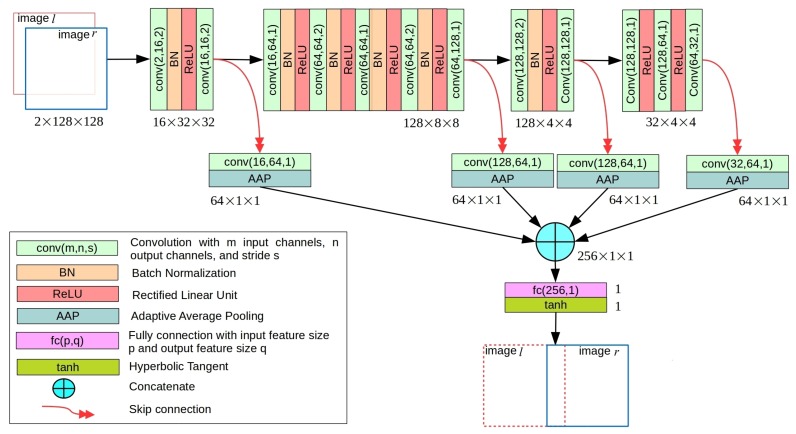
The network architecture of our proposed light-weight ShiftNet, which takes as input two channel-wise stacked 128×128 grayscale images and predicts the relative horizontal shift between the two images. See text for details.

ShiftNet takes as input two channel-wise stacked 128×128 grayscale images extracted from two images $I_{l}$ and $I_{r}$, and predicts the relative horizontal shift between $I_{l}$ and $I_{r}$. The detailed network architecture of ShiftNet is shown in Figure 3. In particular, four convolution blocks composed by a combination of varying number of convolutional layers, batch normalization, and rectified linear unit (ReLU) activation layers are used to extract four feature maps over different scales. Each feature map is converted to a 64D feature vector by passing it through a convolutional layer followed by an adaptive average pooling (AAP) layer, and then the four feature vectors are concatenated together to form a 256D feature vector, which is passed through a subsequent fully-connected layer with one unit. All convolutional layers in ShiftNet are with one pixel of padding. The final output is obtained by applying a hyperbolic tangent activation to the output of the fully-connected layer. In this way, ShiftNet produces a real-valued output within the range [−1,1]. For model training, a $L_{2}$ loss is used. The relative shift between $I_{l}$ and $I_{r}$ is calculated by taking the average of $d_{lr}$ and $d_{rl}$, where $d_{lr}$ and $d_{rl}$ is the outputs of ShiftNet for image pairs $I_{l}↔\;I_{r}$ and $I_{r}↔\;I_{l}$, respectively.

It is noteworthy that the idea of using CNN to solve two-view geometrical problems has been investigated previously in the case of homography estimation [31,32]. While it is straightforward to adopt the existing CNN models for shift estimation by adjusting the output layer only, CNNs based on such modification is not able to obtain sufficiently accurate estimates for cylindrical panorama estimation (as will be shown in the experiments). In comparison, ShiftNet is able to improve the accuracy of recent CNN-based methods [31,32] while maintaining the compactness on model size. Specifically, the model size of ShiftNet is similar to that of the CNN model proposed in [32], which is nearly 30 times smaller than the one proposed in [31]. By gathering and fusing feature maps over multi-scales, ShiftNet is able to capture both global and local features of images and thus achieve robust image alignment with higher accuracy for low-texture scenes.

### 3.4. Global Illumination Invariant Sub-Pixel Refinement

The output of the initial alignment algorithm based on ShiftNet presented in the previous subsection is not accurate enough for cylindrical panorama stitching. A previous study [32] has shown that iterative optimization can achieve higher accuracy for solving similar image registration problem. Motivated by this, we present a global illumination invariant sub-pixel refinement algorithm to further improve the accuracy of the initial estimate under either strict horizontal translation motion or translate and scale motion assumption. The basic idea of sub-pixel refinement is to adjust the motion parameters directly so that the distance between two images is minimized, where the image warping under a certain camera motion model needs to be implemented in a differentiable manner which enables backpropagation of the loss function. The above method has mainly two differences compared with [32], the first is that two different camera motion models for image alignment are used in this paper, the second is that robustness is improved by modeling the global illumination change explicitly in the loss function.

Let $I_{w}$ be the warped image of $I_{l}$ onto $I_{r}$ under a specified camera motion. In order to sample pixels, differential linear (for horizontal translation motion) and bilinear (for translate and scale motion) interpolation is used in this paper. Specifically, in the case of horizontal translation motion, assume that the shift between $I_{l}$ and $I_{r}$ is $\hat{d}$, then the pixel location p=(u,v) in $I_{w}$ is related by the pixel in $I_{l}$ at $q^{ht}=\left(q_{u}^{ht},q_{v}^{ht}\right)=\left(u+\hat{d},v\right)$. In the case of translation and scale motion with horizontal translation $\hat{d_{u}}$, vertical translation $\hat{d_{v}}$, and scale factor $\hat{s}$, the pixel location *p* in $I_{w}$ is related by the pixel in $I_{l}$ at $q^{ts}=\left(q_{u}^{ts},q_{v}^{ts}\right)=\left(\left(u+\hat{d_{u}}\right)/s,\left(v+\hat{d_{v}}\right)/s\right)$. The pixel values of $I_{w}$ at *p* under horizontal translation motion and translation and scale motion are denoted by $I_{w}^{ht}\left(p\right)$ and $I_{w}^{ts}\left(p\right)$, respectively.

**Figure 4:**
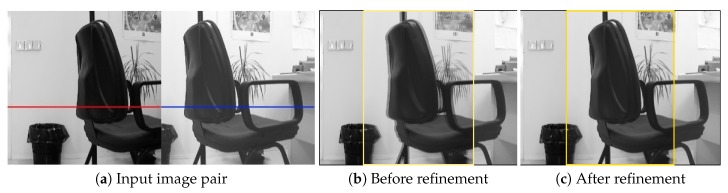
An example of sub-pixel refinement of the horizontal shift between two images ($I_{l}↔I_{r}$) under different illumination conditions. Shown in (**a**) is a pair of input image patches and two highlighted scan lines with the same vertical coordinate. Shown in (**b**,**c**) are the results of image alignment before and after sub-pixel refinement, respectively (improvements of alignment accuracy can be seen from the highlighted overlapping regions).

**Figure 5:**
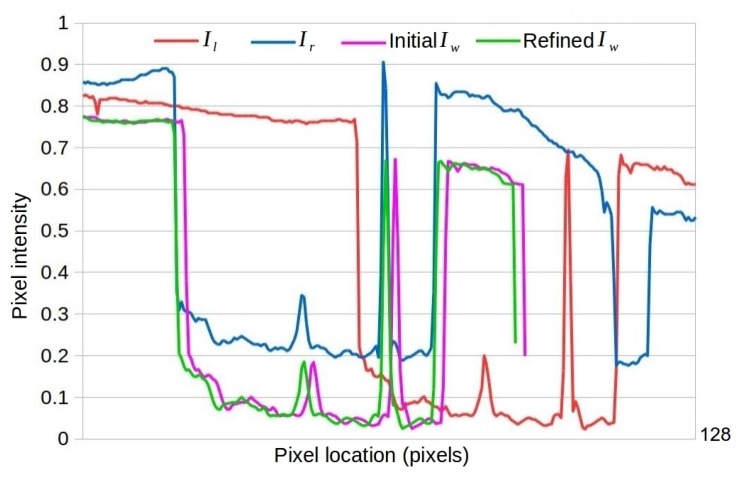
Visualization of the two scan lines in image $I_{l}$ and $I_{r}$ shown in Figure 4a. The scan line of the warped image $I_{w}$ based on the output of ShiftNet (the purple curve) is roughly aligned with that of the destination image $I_{r}$ (the blue curve), and the accuracy of image alignment is improved significantly after applying the proposed sub-pixel refinement (see the alignment between the green curve and the blue curve).

Let $I_{m}$ be a mask image generated by warping a grayscale image with all pixel values equal to one onto $I_{r}$, such that the pixel values within and beyond the valid regions being set to ones and zeros, respectively. The loss function L is defined as

(3) $$L=\sqrt{\frac{\sum_{p\in \Omega }\left\{I_{m}\left(p\right){\left(\alpha I_{w}\left(p\right)-I_{r}\left(p\right)\right)}^{2}\right\}}{\sum_{p\in \Omega }I_{m}\left(p\right)}},$$

where Ω denotes for all pixel locations in image $I_{w}$ and α a scaling factor to be optimized. $I_{w}\left(\cdot \right)$ represents $I_{w}^{ht}\left(\cdot \right)$ or $I_{w}^{ts}\left(\cdot \right)$, depending on the camera motion model used. An illustration of sub-pixel refinement under horizontal translation motion model is shown in Figure 4 and Figure 5.

### 3.5. Synthetic Datasets Generation

In order to train a usable CNN model, a huge amount of data with accurate ground truth labels is essential. Since there is no available public dataset for the task of image alignment with ShiftNet, we generate pairs of image patches with random horizontal shifts based on background images randomly selected from the Microsoft common objects in context (MS-COCO) dataset [42], which consists of a training dataset with 82,783 images, a validation dataset with 40,504 images, and a test dataset with 40,775 images. In our experiments, 1,000,000 training image patch pairs, 10,000 validation image patch pairs, and 10,000 test image patch pairs are generated from the training dataset, validation dataset, and the test dataset, respectively.

**Figure 6:**
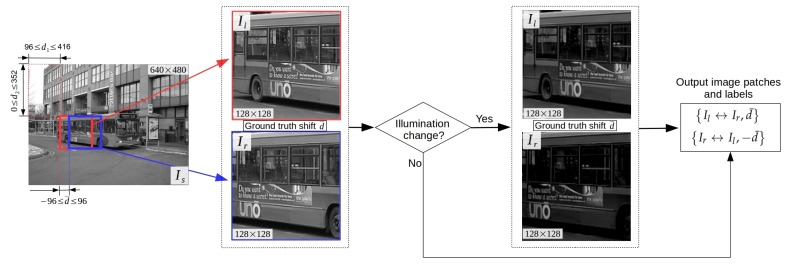
The workflow for generating synthetic image patches from the publicly available COCO dataset [42]. In each iteration, two pairs of image patches with random illumination changes are generated. See text for details.

**Figure 7:**
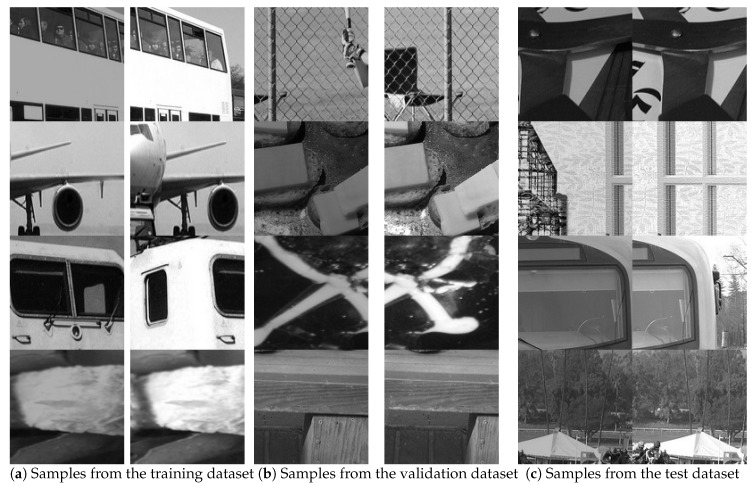
Image patch samples from our generated synthetic datasets with large diversity in the amount of displacements, illumination conditions, and scene types: (**a**) training dataset; (**b**) validation dataset; and (**c**) test dataset.

The workflow for generating synthetic image patches is illustrated in Figure 6. Specifically, a source image $I_{s}$ is randomly selected from MS-COCO training (or validation, test) dataset and resized to 640×480 pixels. Then, two image patches $I_{l}$ and $I_{r}$ of size 128×128 pixels ared obtained by extracting the rectangular subregions of $I_{s}$ starting at point $\left(d_{1},d_{2}\right)$ and $\left(d_{1}+\bar{d},d_{2}\right)\left(96\leq d_{1}\leq 416,0\leq d_{2}\leq 352\right)$, respectively. In this way, the range of ground truth horizontal shift $\bar{d}$ is set to [−96,96], which is 3/4 of the size of image patch. Data augmentation strategies are adopted to enhance the diversity of dataset. First, to compensate for global illumination change, the brightness of a pair of image patches is scaled down with probability of 50% by a random factor within 0.6 and 1.0. The other augmentation exploiting the symmetry in data is done by switching the order of image patches. Thus, two pairs of image patches with corresponding ground truth labels ($\left\{I_{l}↔I_{r},\bar{d}\right\}$ and $\left\{I_{r}↔I_{l},-\bar{d}\right\}$) can be generated. The resulting synthetic datasets are of large diversity in the amount of displacements, illumination conditions, and scene types, which is helpful for training a robust CNN model. A few image samples from our generated training, validation, and test sets are shown in Figure 7.

## 4. Experiments

In order to evaluate the performance of our proposed method, we implemented the algorithms described in Section 3. The test machine used in our experiments is equipped with an Intel(R) Core(TM) i7-7700HQ CPU at 2.80 GHz (with four cores and eight threads), a NVIDIA GeForce GTX 1060 GPU, and 16 GB physical memory. The operation system for our test machine is Ubuntu 16.04. Both qualitative and quantitative evaluation on synthetic data, rendered photo-realistic images, and real images were conducted and compared with existing feature based methods and deep learning based methods, which enables thorough examination of the performance of different methods.

### 4.1. Baseline Methods

The five comparative feature based methods are based on popular sparse local image features including SIFT, SURF, ORB, AKAZE, and BRISK. For this set of methods, the particular image feature detector is used to detect points of interest. Putative keypoint correspondences are established by nearest neighbor search via fast library for approximate nearest neighbors (FLANN) followed by a ratio test proposed by Lowe [12]. Outliers are filtered out further by checking the difference of the coordinates along the vertical direction (which we call shift test in the following). The final estimate of shift is obtained by taking the median of the horizontal coordinate differences extracted from all feature correspondences for robust estimation. All the above five methods are implemented in OpenCV with default parameter settings and the threshold for ratio test and shift test is set to 0.75 and 5.0 pixels, respectively. Image alignment failure occurs if there is no correctly identified feature correspondence. In the following, the above comparative feature based methods are denoted by sift, surf, orb, akaze, and brisk, respectively.

As for deep learning based methods, we compare our proposed ShiftNet with algorithms based on CNN models described in [31,32]. Note that since both [31,32] aim at estimating homography between images, the final fully connected output layer of the CNN models is modified accordingly to output a single value for the purpose of image shift estimation, and the resulting CNN models are denoted by shiftvgg and shiftvggsim, respectively. Our proposed ShiftNet, shiftvgg, and shiftvggsim are all used for initial alignment, which can be further refined with sub-pixel refinement procedures, either under horizontal translation (HT) motion assumption or translation and scale (TS) transformation assumption as described in Section 3.4. In the following, we use the suffix “_ht” and “_ts” to denote for refinement based on HT and TS model, respectively. In this way, nine deep learning based methods denoted by shiftnet, shiftnet_ht, shiftnet_ts, shiftvgg, shiftvgg_ht, shiftvgg_ts, shiftvggsim, shiftvggsim_ht, and shiftvggsim_ts are evaluated in our experiments.

The three CNN models shiftnet, shiftvgg, and shiftvggsim are all implemented in PyTorch and the Adam optimizer [43] with momentum 0.9 is used for optimization. The models are trained on a NVIDIA TITAN X GPU with 12 GB memory in an end-to-end fashion from scratch for 100 epochs, where all weights are initialized randomly. For shiftnet and shiftvggsim, the batch size for training is set to 2048 and the initial learning rate is set to $1\times 10^{-3}$. For shiftvgg, a batch size of 32 was used due to limits of GPU memory, and an initial learning of 0.001 is used for numerical stability. For the training of all the three models, the learning rate is scaled by a factor of 0.1 if the decrease of loss value within an epoch is below $1\times 10^{-7}$. Sub-pixel refinement procedures are also implemented in PyTorch, and a fixed learning rate 0.001 is used for optimization.

### 4.2. Experiments on Synthetic Datasets

We first evaluate the performance of various methods on the synthetic test dataset which was generated by a way described in Section 3.5. The test dataset contains 10,000 image pairs with ground truth horizontal shifts, which enables both qualitative and quantitative evaluation.

**Figure 8:**
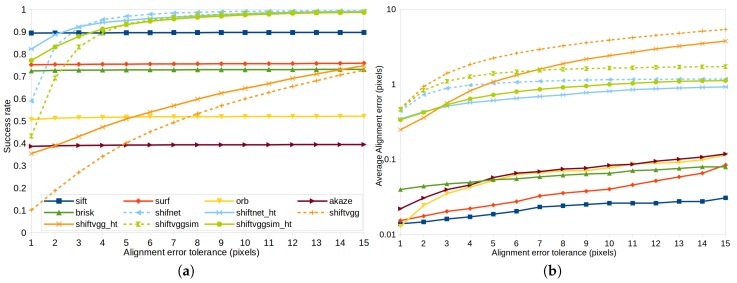
Comparison of image alignment performance on the synthetic test dataset (with 10,000 images) using different methods. The results in (**a**,**b**) are the success rate and average alignment error under varying alignment error tolerance, respectively.

Shown in Figure 8 are the results of the five feature based methods and six deep learning based methods including shiftnet, shiftnet_ht, shiftvgg, shiftvgg_ht, shiftvggsim, and shiftvggsim_ht. The other three deep learning based methods are not tested on this test dataset because it involves purely horizontal shift only. In particular, Figure 8a shows the success rate of different methods under increasing error tolerance. Feature based methods fail when the alignment error (the absolute distance between the estimated shift and the corresponding ground truth value) is above the specified error tolerance or no feature correspondence can be established due to lack of texture. In other words, feature based methods are expected to work if only feature correspondences can be found between images. Figure 8a also shows that among all the tested feature detection methods, sift is the most robust one which achieves an overall of 90% success rate (i.e., sift fails to establish feature correspondences for 10% of the tested image pairs), while akaze performs the worst whose overall success rate is less than 40%. The success rate of surf, brisk, and orb is about 75%, 72%, and 51%, respectively. From the results, it is easy to see that the error tolerance has nearly no impact on the success rate of the five feature based methods, which indicates that the localization accuracy of local image features is high and the failure of these methods is mainly due to texture-less scene objects. As for deep learning based methods, the results in Figure 8a demonstrate that our proposed shiftnet and shiftnet_ht perform consistently better than all comparative methods in all cases for any tolerance values greater than 2.8 pixels. The experimental results also show that the use of sub-pixel refinement improves the success rate significantly in a majority of cases especially for smaller error tolerance.

The experimental results in Figure 8b show the average alignment error of different methods under increasing error tolerance. In most cases, the feature based methods achieve an average alignment error below 0.1 pixels, which is extremely low mainly because the image pairs in the ideal test dataset are under strict pixel-level horizontal translation motion and no distortion, noise, or re-sampling artifacts are added to the image, thus the alignment error actually depends only on the localization accuracy of keypoint detection itself. For deep learning methods, sub-pixel refinement improves the accuracy of estimation significantly in all cases. shiftnet_ht achieves the highest accuracy among all deep learning based methods for any error tolerance greater than 2.8 pixels. It is noteworthy that although the accuracy of shiftvgg_ht is higher than that of shiftnet_ht for an error tolerance below 1.6 pixels, the success rate of shiftvgg_ht is significantly lower than that of shiftnet_ht (see Figure 8a), which means that shiftvgg_ht is able to obtain highly accurate shift estimates only for a small number of image pairs.

**Figure 9:**
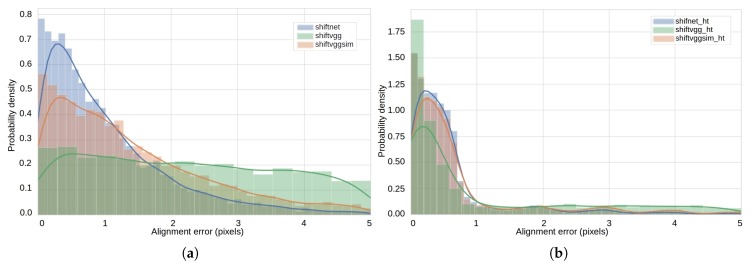
Statistics of image alignment error: (**a**) the distribution of alignment error on the synthetic test dataset using different methods and (**b**) the corresponding probability density fitted using Gaussian kernel density estimation (KDE).

To facilitate in-depth investigation, the distribution of average alignment errors (error larger than 5.0 pixels is not shown) with an error tolerance of 5.0 pixels for three deep learning based methods without and with sub-pixel refinement is shown in Figure 9a,b, respectively. The solid lines in Figure 9 are the fitted probability density function calculated using Gaussian kernel density estimation (KDE) algorithm [44]. The statistical graphics in Figure 9 are visualized with seaborn, which is a high-level Python data visualization library based on matplotlib. From Figure 9a, we can see that the probability density of average alignment errors below 1.0 pixels for shiftnet is considerably larger than the comparative methods, which is also true for shiftnet_ht as shown in Figure 9b. By comparing the experimental results in Figure 9a,b, the superiority of employing the sub-pixel refinement can also be easily observed.

**Figure 10:**
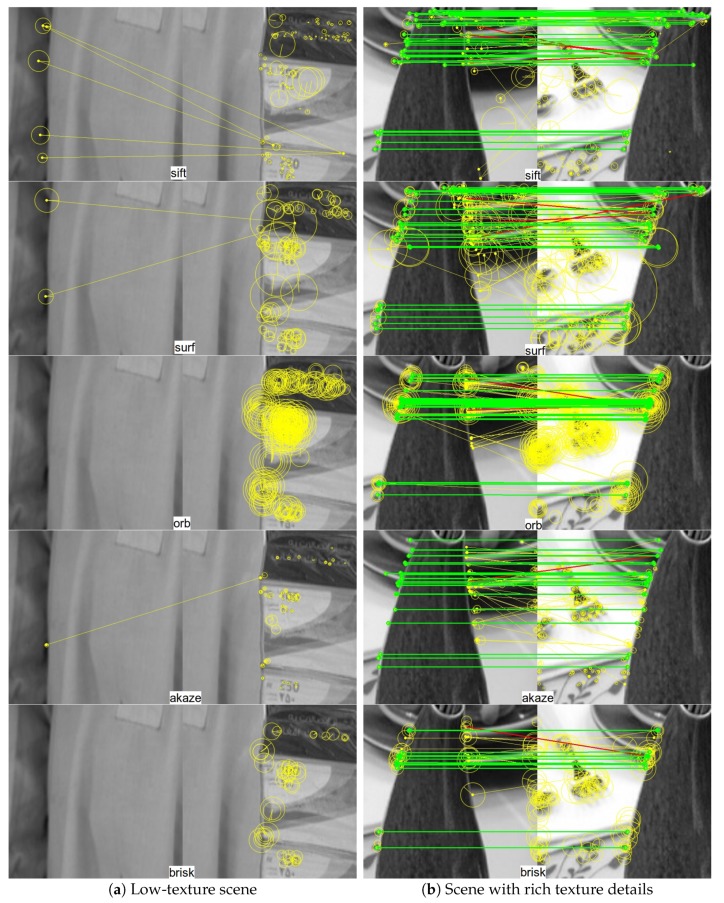
Qualitative evaluation of different feature based image alignment methods on two pairs of synthetic test image patch samples: (**a**) low-texture scenes; (**b**) scenes with rich texture details. Each detected keypoint are shown as a circle around it with keypoint size and orientation visualized. A keypoint and its nearest neighbor found by fast library for approximate nearest neighbors (FLANN) are connected with a yellow line segment. Putative keypoint correspondences passing both the ratio test and shift test are connected with green line segments, while keypoints passing the ratio test but fail the shift test are higlighted with red line segments. From top to bottom are the results of sift, surf, orb, akaze, and brisk, respectively. See text for details.

**Figure 11:**
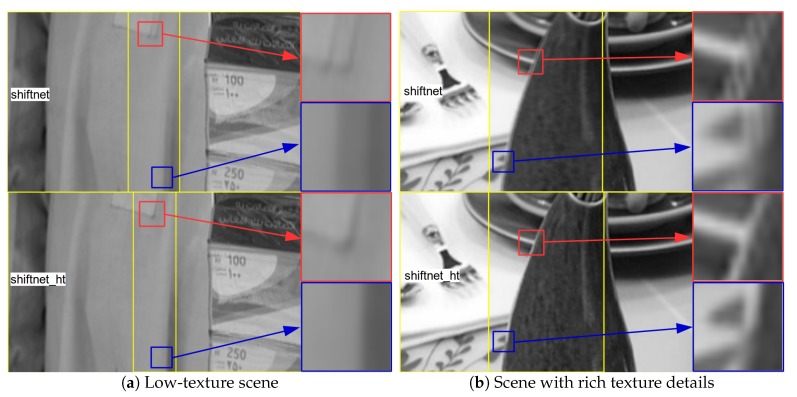
Results of image alignment (with interested regions highlighted and zoomed in) for the same image pairs as shown in Figure 10. For both image pairs, the stitched images in the upper and lower part are the results of shiftnet and shiftnet_ht, respectively. See text for details.

To facilitate visual inspection, representative qualitative results are shown in Figure 10 and Figure 11. In particular, Figure 10 presents the results of feature detection and matching on two image pairs using different feature based methods, where Figure 10a corresponds to a scene with low texture and Figure 10b shows a scene with fine details. From top to bottom are the results of sift, surf, orb, akaze, and brisk, respectively. For each keypoint, the circle around keypoint with keypoint size and orientation are visualized. A keypoint and its nearest neighbor found by FLANN are connected with a yellow line segment. Putative keypoint correspondences passing both the ratio test and shift test are connected with green line segments, while keypoints passing the ratio test but failing the shift test are higlighted with red line segments. For the texture-less image pair shown in Figure 10a, none of the tested sparse feature detection and matching method is able to establish any correct keypoint correspondence, which leads to failure in subsequent image alignment process. In comparison, for the image pair shown in Figure 10b, all five feature based methods succeed in establishing a number of good feature correspondences and rejecting wrong matches, based on which image alignment with high accuracy can be accomplished. As already shown in Figure 8, the success rate of image alignment using different feature based methods ranges from around 40% to 90% for this synthetic test dataset.

Figure 11 presents the image alignment results for the same image pairs as shown in Figure 10. For both image pairs, the stitched images in the upper and lower part are the results of shiftnet and shiftnet_ht, respectively. Interested regions are highlighted and zoomed in in order to view the details of stitched images. From Figure 11 it is easy to see that the amount of visual artifacts reduce noticeably after with the use of sub-pixel refinement, which demonstrates that shiftnet is able to provide sufficiently good initialization for sub-pixel refinement and shiftnet_ht achieves significantly higher accuracy. The above results coincide with the results in Figure 8b. Moreover, the results shown in Figure 11 also suggest superior performance over feature based method regarding robustness.

### 4.3. Experiments on Rendered Photo-Realistic Images

**Figure 12:**
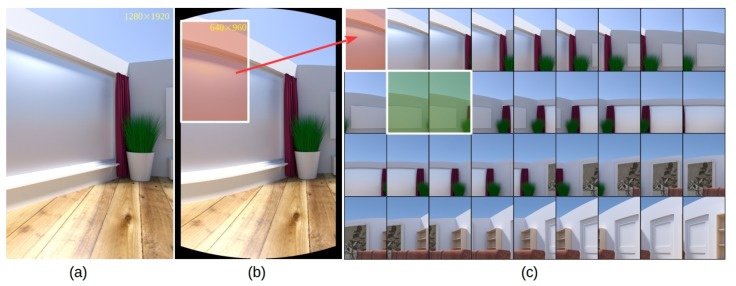
Construction of a challenging rendered photo-realistic image sequence for testing cylindrical panorama stitching methods: (**a**) an example source image [40]; (**b**) the corresponding cylindrical projection of (**a**); (**c**) the resulting image sequence used in our experiments consisting of 36 images.

Next, we investigate the performance of different methods for the task of cylindrical panorama generation. To this end, experiments on a challenging image sequence generated from a publicly available synthetic dataset [40] were carried out. The original synthetic dataset consists of 72 images (the resolution of image is 1280×1920 pixels) of a virtual sparsely structured 3D scene rendered with Blender by rotating the camera around a fixed axis passing through its optical center exactly 5 degrees between consecutive images. Since the intrinsic parameters of the the virtual camera is available, cylindric projection of the original image can be fulfilled by following the method described in Section 3.2. One of the original rendered photo-realistic images and its cylindrical projection the are shown in Figure 12a,b, respectively. Instead of using the whole dataset as input, we construct a more challenging dataset by extracting a region from a cylindrical projected image as shown in Figure 12b. The generated final photo-realistic image sequence used in our experiments consists of 36 images of 640×960 pixels and the thumbnails for all images are shown in Figure 12c.

**Figure 13:**
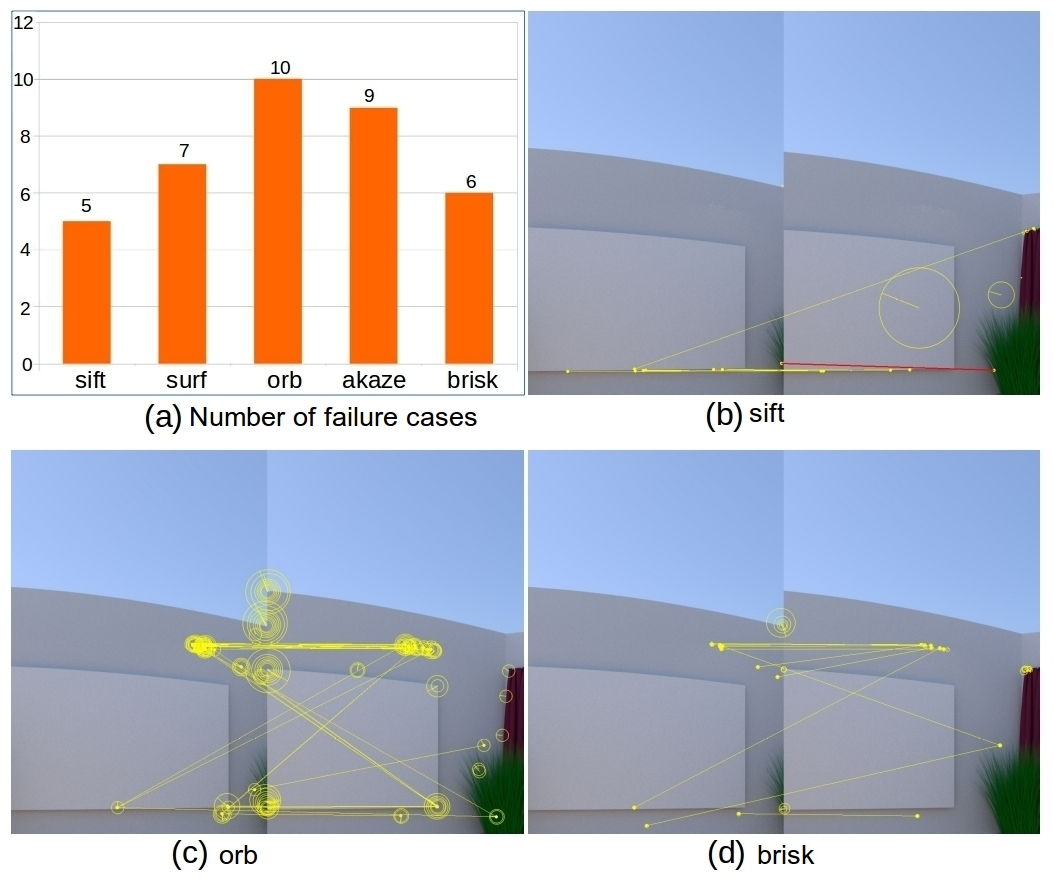
Experimental results of feature based image alignment on rendered photo-realistic image sequence. Shown in (**a**) is the number of failure cases out of 35 image pairs. Shown in (**b**–**d**) are the results of image matching for the image pair highlighted in green in Figure 12c using sift, orb, and brisk, respectively.

In order to fulfill panorama stitching for the above image sequence, there are 35 consecutive image pairs that need to be aligned and alignment failure in any of these 35 image pairs will lead to failure in the cylindrical panorama generation. Since the scene is texture-less for many images (see Figure 12c), image alignment based on sparse feature detection and a matching algorithm is not applicable due to failure in image matching. A statistics of failure cases for the tested image sequence and image matching results for the image pair highlighted in green in Figure 12c are shown in Figure 13. In particular, Figure 13a presents the number of failure cases out of all 35 image pairs for sift, surf, orb, akaze, and brisk. The statistics shows that failure rate for feature based methods on this image sequence ranges from 14.29% to 28.57%, among which sift performs best and orb is the least robust one. The results of feature detection and matching with sift, orb, and brisk are presented in Figure 13b–d, respectively (surf and akaze fail to detect feature points in at least one image thus omitted). The visualization of feature points and correspondences follows the same way as that in Figure 10. From the experimental results, we can see that sift, orb, and brisk are able to detect and identify a set of putative keypoint correspondences for this pair of images (see Figure 13b–d). However, none of the tested feature based methods are able to establish any keypoint correspondence passing both the ratio test and horizontal test, which leads to failure in image alignment and thus the subsequent cylindrical panorama stitching.

Since the motion of the virtual camera is rotated under precise control while rendering images, the ground truth translation between consecutive images can be calculated easily, making it possible to evaluate the alignment algorithms quantitatively. The average accuracy of image alignment on the 35 image pairs using six deep learning based methods are shown in Table 1, from which we can see that our proposed shiftnet model is able to obtain significantly better results than both shiftvgg and shiftvggsim models, and that sub-pixel refinement improves alignment accuracy significantly in most cases. The above observations coincide with that of synthetic test dataset (see Figure 8b).

**Figure 14:**
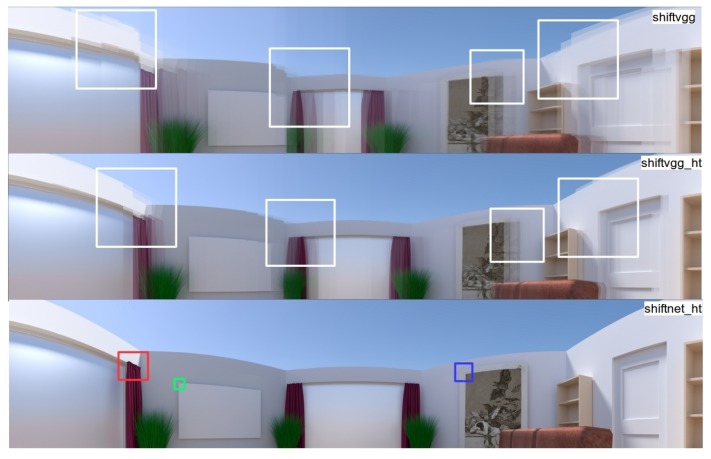
Qualitative comparison of the generated cylindrical panoramas from the constructed rendered photo-realistic image sequence using different deep learning based methods. From top to bottom are results of shiftvgg, shiftvgg_ht, and shiftnet_ht, respectively (interested regions are highlighted). See text for details.

More qualitative comparison of the generated cylindrical panoramas using different deep learning based methods are shown in Figure 14, where the results of shiftvgg, shiftvgg_ht, and shiftnet_ht are shown in the first, second, and the third row, respectively. While shiftvgg_ht generates panorama with higher quality than shiftvgg, both shiftvgg and shiftvgg_ht result in noticeable artifacts due to inaccurate shift estimation (see the highlighted regions in the first two rows in Figure 14). In comparison, as shown in the last row of Figure 14, our proposed shiftnet_ht is able to generate a much more visually pleasing panorama.

**Figure 15:**
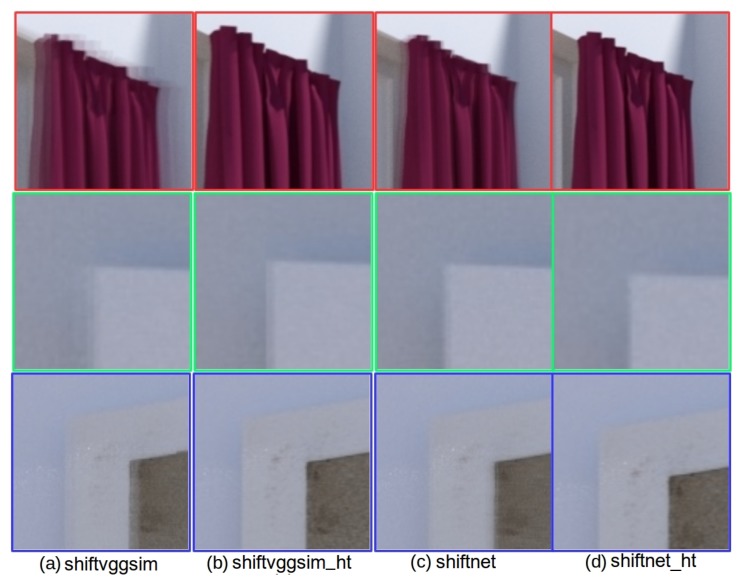
Qualitative comparison of enlarged views corresponding to panoramas generated with different methods for the three highlighted regions as shown in the last row of Figure 14: (**a**) shiftvggsim, (**b**) shiftvggsim_ht, (**c**) shiftnet, and (**d**) shifnet_ht. See text for details.

Since shiftnet, shiftvggsim, and shiftvggsim_ht are all able to produce results hard to compare from the global view, thus we present in Figure 15, a comparison of zoomed-in views corresponding to panoramas generated with different methods for the three highlighted regions as shown in the last row of Figure 14. In particular, the results of shiftvggsim, shiftvggsim_ht, shiftnet, and shiftnet_ht are presented in Figure 15a–d, respectively. Again, the experimental results suggest that sub-pixel refinement is able to improve alignment accuracy noticeably and shiftnet_ht achieves the best overall performance.

### 4.4. Experiments on Real Images

**Figure 16:**
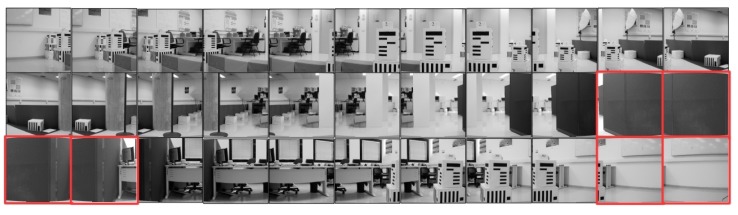
Real image sequence used in our experiment taken from [6]. Images of low-texture scenes which pose challenge for image alignment using feature based methods are highlighted.

In order to test the robustness of different methods in practical scenario, we carried experiments on a public available real image sequence with 33 479×480 images captured by a Sony DFW-VL500 camera mounted on a directed perception PTU-46-70 pan-tilt unit [6]. The thumbnails for the cylindric projected images are shown in Figure 16, where the six highlighted images are selected for qualitative evaluation of feature based methods due to lack of texture.

**Figure 17:**
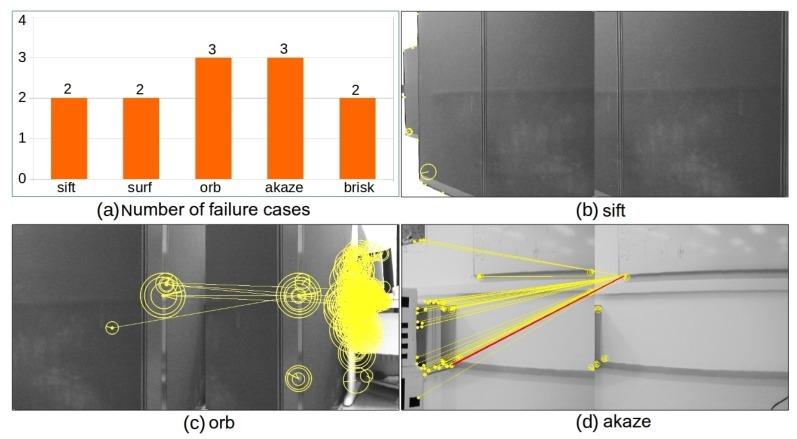
Experimental results of feature based image alignment on real image sequence. Shown in (**a**) is the number of failure cases out of 32 image pairs. Shown in (**b**–**d**) are example results of image matching using sift, orb, and akaze, respectively (the visualization follows the same strategy as that in Figure 10, except that the brightness of figures is adjusted to facilitate visual inspection).

In the image matching process, all methods in sift, surf, orb, akaze, and brisk fail to establish good feature correspondences for at least two image pairs. The number of failure cases for each method is presented in Figure 17a. The results of feature detection and matching with sift, orb, and akaze for selected challenging images are presented in Figure 17b–d, respectively (surf and brisk get similar results, and are thus omitted). The visualization of feature points and correspondences follows the same as that in Figure 10. From the experimental results we can see that orb and akaze are able to detect and identify a set of putative keypoint correspondences (see Figure 17c,d). However, none of the tested feature based methods are able to establish any keypoint correspondence passing both the ratio test and shift test for the above images, which leads to failure in image alignment and thus the subsequent cylindrical panorama stitching.

**Figure 18:**
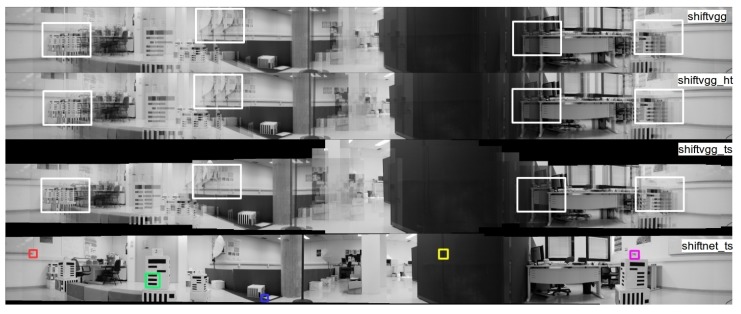
Qualitative comparison of the generated cylindrical panoramas from real images using different deep learning based methods. From top to bottom are the results of shiftvgg, shiftvgg_ht, shiftvgg_ts, and shiftnet_ts, respectively (interested regions are highlighted). See text for details.

Qualitative comparison of the generated cylindrical panoramas from the real image sequence using different deep learning based methods are shown in Figure 18, where from top to bottom are the results of shiftvgg, shiftvgg_ht, shiftvgg_ts, and shiftnet_ts, respectively. As can be seen from the first three rows in Figure 18 (see the highlighted regions), both shiftvgg and its variants (shiftvgg_ht and shiftvgg_ts) introduce severe artifacts in the stitched cylindrical panoramas, mainly due to poor initialization provided by shiftvgg. In comparison, as shown in the last row of Figure 18, our proposed shiftnet_ts is able to generate a much more visually pleasing panorama, in which five regions are highlighted for further inspection.

**Figure 19:**
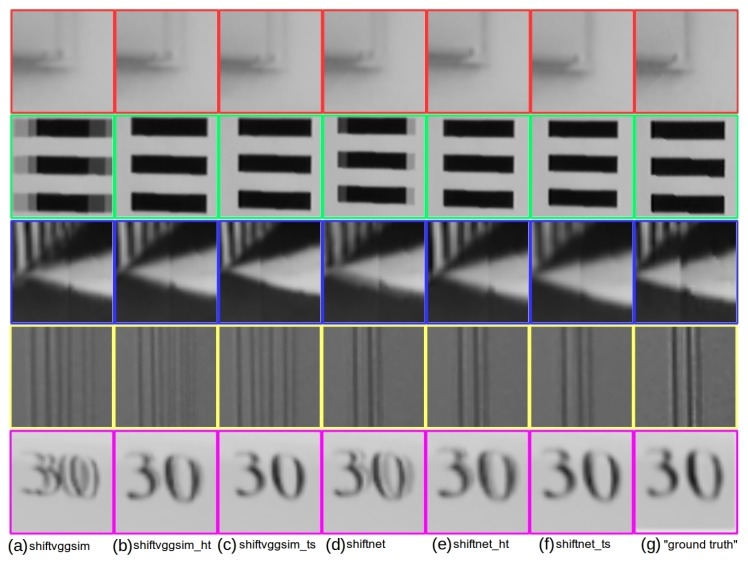
Qualitative comparison of zoomed-in views corresponding to panoramas generated with different methods for the three highlighted regions as shown in the last row of Figure 18: (**a**) shiftvggsim, (**b**) shiftvggsim_ht, (**c**) shiftvggsim_ts, (**d**) shiftnet, (**e**) shifnet_ht, (**f**) shiftnet_ts, and (**g**) reference “ground truth”.

Not all the whole panoramas generated by all the deep learning based methods are presented, as they produce results hard to compare from the global view. Instead, in Figure 19 we present a comparison of enlarged views corresponding to panoramas generated with different deep learning based methods for the five highlighted regions as shown in the last row of Figure 18. In particular, Figure 19a–f are the results of shiftvggsim, shiftvggsim_ht, shiftvggsim_ts, shiftnet, shiftnet_ht, and shiftnet_ts. Regions of interests from the “ground truth” panorama provided by [6] is shown in Figure 19g for reference. The experimental results confirm that sub-pixel refinement under horizontal translation assumption is able to improve alignment accuracy noticeably, and alignment accuracy can be further increased with sub-pixel refinement under translation and scale transformation assumption. While shiftnet_ts achieves the best overall performance, noticeable artifacts can be found in at least one region for any of the comparative methods (shiftvggsim, shiftvggsim_ht, shiftvggsim_ts, shiftnet, shiftnet_ht). It is also noteworthy that shiftnet_ts generates better results than the provided “ground truth” based on strict horizontal translation motion assumption for some regions (see the second and third rows in Figure 19f,g, which suggests that our proposed motion compensation is necessary in real applications, where camera motion does not undergo ideal rotation with respect to a fixed axis passing through its optical center.

### 4.5. Comparison with Existing Software

**Figure 20:**
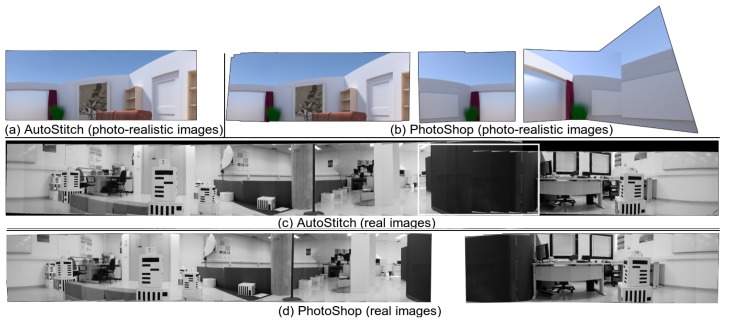
Comparison of cylindrical panorama using existing software. See text for details.

In addition, we made a comparison of cylindrical panorama stitching using the popular free demo AutoStitch [10] and commercial software PhotoShop. The experimental results are presented in Figure 20, where the images shown in Figure 20a,b are the results on the rendered photo-realistic image sequence shown in Figure 12c using AutoStitch and PhotoShop, respectively. The images shown in Figure 20c,d are the results on the real image sequence shown in Figure 16 using AutoStitch and PhotoShop, respectively. From the experimental results, it is easy to see that the existing tools failed to construct complete and visually pleasing panoramas for both challenging image sequences.

## 5. Conclusions

In the process of cylindrical panorama generation, traditional image stitching methods based on hand-crafted features are unable to handle low-texture environments where no reliable feature correspondence can be established. In order to address the above limitation, a novel two-step image alignment method based on deep learning and iterative optimization is proposed. Specifically, an end-to-end trainable light-weight CNN architecture called ShiftNet is proposed to estimate the initial shifts between images, which is further optimized in a sub-pixel refinement processing based on a specified camera motion model. We carried out extensive experiments on a synthetic dataset, on rendered photo-realistic images, and on real images to evaluate the performance of our proposed and comparative methods. The qualitative and quantitative experimental results demonstrate that cylindrical panorama stitching based on our proposed image alignment method leads to significant improvements over traditional feature based methods and recent deep learning based methods for challenging low-texture environments. The use of deep learning based image alignment in many other potential applications such as satellite image stitching, multi-spectral satellite image registration, and environmental modeling and localization are directions worthy of further investigation.

## Figures and Tables

**Table 1:** Accuracy of image alignment on the rendered photo-realistic image sequence using different methods. Best and second best results are highlighted in bold.

Methods	Mean	Std. Dev	Median	Minimal	Maximum
shiftnet	**3.604**	4.140	3.805	0.203	**15.904**
shiftnet_ht	**1.517**	**1.531**	**1.274**	**0.090**	**7.487**
shiftvgg	38.236	48.473	38.947	0.210	134.098
shiftvgg_ht	22.473	20.354	13.504	**0.027**	79.191
shiftvggsim	6.007	6.528	5.785	0.185	35.968
shiftvggsim_ht	6.153	**2.530**	**1.272**	0.091	35.532
